# Field Detection of Rhizoctonia Root Rot in Sugar Beet by Near Infrared Spectrometry

**DOI:** 10.3390/s21238068

**Published:** 2021-12-02

**Authors:** Leilane C. Barreto, Rosa Martínez-Arias, Axel Schechert

**Affiliations:** Strube Research GmbH & Co., KG, Neue Strasse 11, 38838 Schlanstedt, Germany; r.martinez@strube-research.net (R.M.-A.); A.Schechert@strube.net (A.S.)

**Keywords:** *Rhizoctonia solani*, near-infrared spectroscopy, soil-borne pathogen, disease detection, *Beta vulgaris*

## Abstract

Rhizoctonia root and crown rot (RRCR) is an important disease in sugar beet production areas, whose assessment and control are still challenging. Therefore, breeding for resistance is the most practical way to manage it. Although the use of spectroscopy methods has proven to be a useful tool to detect soil-borne pathogens through leaves reflectance, no study has been carried out so far applying near-infrared spectroscopy (NIRS) directly in the beets. We aimed to use NIRS on sugar beet root pulp to detect and quantify RRCR in the field, in parallel to the harvest process. For the construction of the calibration model, mainly beets from the field with natural RRCR infestation were used. To enrich the model, artificially inoculated beets were added. The model was developed based on Partial Least Squares Regression. The optimized model reached a Pearson correlation coefficient (R) of 0.972 and a Ratio of Prediction to Deviation (RPD) of 4.131. The prediction of the independent validation set showed a high correlation coefficient (R = 0.963) and a root mean square error of prediction (RMSEP) of 0.494. These results indicate that NIRS could be a helpful tool in the assessment of Rhizoctonia disease in the field.

## 1. Introduction

Rhizoctonia root and crown rot (RRCR) is a widespread disease in sugar beet (*Beta vulgaris* L.) production fields which has been disseminating over a large area in Europe and in the United States in the last decade [1,2,3]. RRCR is caused by the soil-borne fungus *Rhizoctonia solani* J. G. Kühn, a pathogen that lives in soil independently of the host presence, competes with the microflora and depends on the host plant and on the environment to propagate over space and time [4,5].

RRCR is usually associated with the developing canopy and occurs mostly late in the cropping season on older plants [6]. After the invasion of the fungus on petioles in contact with the soil, black lesions appear on the base, then the rotting progresses to the crowns and roots accompanied by above-ground symptoms that include severe wilting, collapse and yellowing of leaves. Root rot develops as brown to black, sunken and circular lesions, which often clump together and cover large areas of the root surface [5,7]. The affected and stunted plants form elongated patches that are dynamic and vary in size in the fields, changing the disease pattern in the affected areas within and between seasons [4].

The yield loss caused by Rhizoctonia infection is determined by the extent of the disease attack and varies from field to field, reaching up to a 60% yield depletion [8,9]. The unpredictable occurrence of the disease and the varying intensity of losses in different seasons make the control of RRCR in sugar beet extremely difficult, resulting in an inherent epidemiological variability and uncertainty to the producers about the severity of the disease in a given season. In addition, this disease also affects beet storage and the technical quality of sugar beets, both factors playing an important role during sugar processing and extraction [1,3,4].

Controlling the RRCR disease is a challenge for breeders and farmers, and so far there is still no effective, economically practical and environmentally safe method to restrict disease outbreaks [3,4]. The attempts of disease management of *R. solani* on sugar beet include crop rotation, fungicide application with recommended rates during seed treatment, as well as in 6–8 leaf stage in some countries, and the development of resistant cultivars [2,9]. Therefore, breeding for resistance to *R. solani* has been a must and an on-going work for sugar beet breeders in the last decades, offering the most practical way to control the disease. However, immunity to *R. solani* has not been found yet in sugar beet germplasm. The incorporation of high levels of resistance in commercial cultivars takes many years, and breeding for resistance requires reliable methods for disease assessment [1,2,10].

To evaluate the severity of the disease, breeders usually rely on visual evaluation of surface rot on beets under greenhouse artificial inoculation. Within a field, a disease lesions assessment in the beets might be a more direct and precise method than in the greenhouse. Nevertheless, this assessment is destructive and entails a limited sampling size, besides the fact that visual disease assessments are subjective and can be therefore biased [11]. Indeed, and according to Büttner et al. [1], one of the limitations that breeders face when trying to select for Rhizoctonia resistant genotypes, is the lack of suitable methods for disease assessment. Several studies have tried to assess disease severity by using spectroscopy methods in sugar beet leaves. Hillnhütter et al. [12,13] demonstrated that the symptoms caused by RRCR induced modifications that could be detected by hyperspectral image analysis. When investigating the potential of remote sensing in the early detection of RRCR in sugar beet, Reynolds et al. [14] concluded that remote sensing can detect the disease, but only after the initial appearance of foliar symptoms. Barreto et al. [15] as well as Strube GmbH & Co. [16] showed that under controlled conditions the early detection of indirect symptoms caused by Rhizoctonia root rot in sugar beet plants is possible using leaves reflectance information. However, when comparing the scoring of leaf symptoms with the scoring based on root symptoms, Scholten et al. [10] concluded that it is not recommendable to use leaf symptoms for disease assessment, as they might be more erratic. Considering that, in this study we applied near-infrared spectroscopy (NIRS) directly on sugar beet roots. Based on specific signatures of electromagnetic radiation absorbance in the NIR range, it is well known that NIRS provides a powerful while simple and rapid analytical technique with little sample preparation time and high-throughput [17,18,19]. And thus, precision agriculture has greatly benefited from a fast and continuous development of NIRS prediction models in the last decades. In sugar beet, NIRS applications have so far focused their efforts on root performance and quality traits [20,21,22,23,24]. This study aimed to expand those applications to the detection and quantification of root diseases, by developing a fast and reliable prediction of RRCR incidence scoring that could be applied directly in the field.

## 2. Materials and Methods

### 2.1. Field Trials

**Figure 1:**
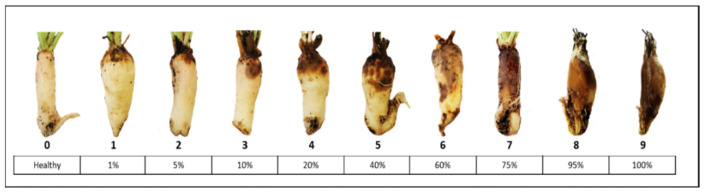
Disease scale used to score Rhizoctonia root and crown rot in sugar beet based on the percentage of rotten root surface area.

**Figure 2:**
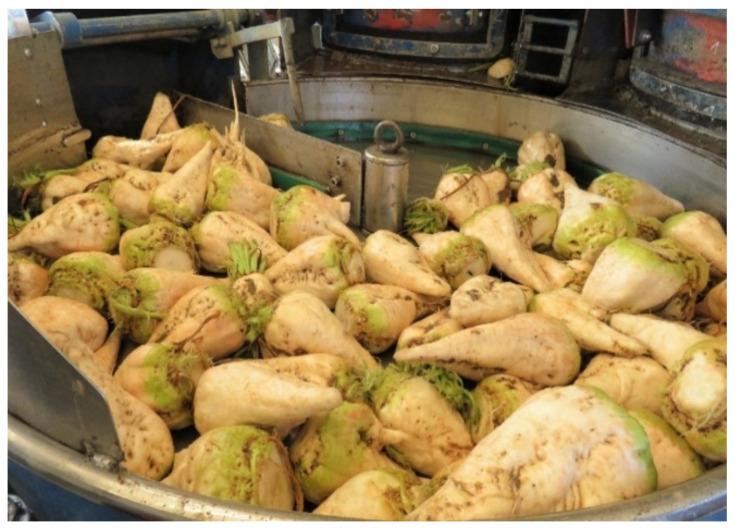
Sugar beet beets after the washing step and at the time of scoring RRCR in the harvester.

An alpha lattice design layout with three row plots of 9 m^2^ was used in the field trials. Each trial plot consisted of 90 sugar beets, which were harvested, topped, washed, ground and finally measured with NIR. After the washing step, the beets were clean and spread (Figure 1) and could be scored for Rhizoctonia infection. The scoring of disease incidence was performed taking into account both the number of affected beets per plot and the severity of the infection, which was based on the percentage of rotten root surface in relation to the volume of the beet. For this visual assessment in the field, the beets were rated based on a disease classes scale modified from Büttner et al. [1] ranging from 0 to 9, where 0 indicates healthy beets and 9 highly infected (Figure 1). All beets from a given plot were scored at once (Figure 2).

**Figure 3:**
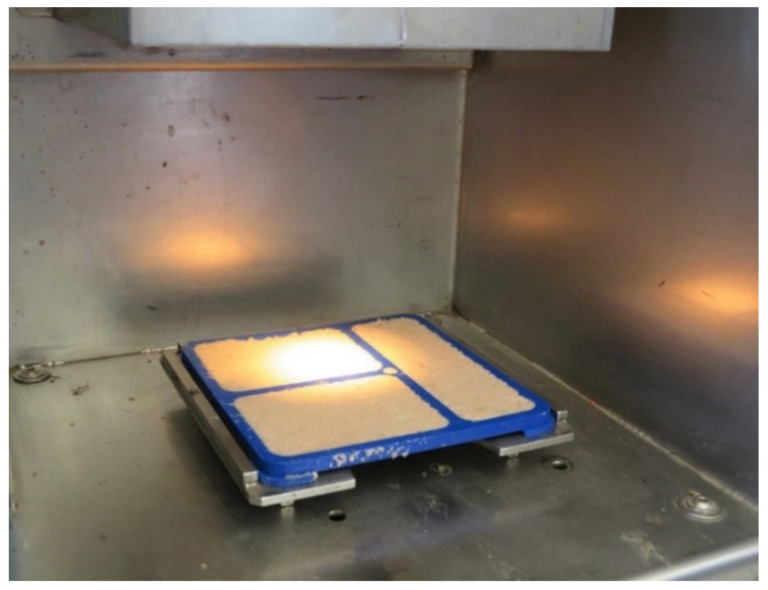
Homogeneous and representative pulp samples from a given field plot being measured with NIR under reflectance. NIR equipment was installed in the harvester.

Thereafter, all beets per plot were ground into a very fine pulp, which was then well mixed and spread in 7-mm-thick plates that were immediately measured by NIRS (Figure 3). One measurement was taken therefore from a homogeneous pulp sample, representative for the 90 beets of a given plot. Consequently, the samples in this study are to be considered as individual samples and not replicates. NIR measurements were integrated to the harvesters and taken in parallel to the harvest process in the field. The spectrometer PSS 1720 (Polytec GmbH, Waldbronn, Germany) was used to collect spectra under reflection. Spectral information was recorded every 2 nm in the spectral range 850–1650 nm. A single NIR measurement took 3.2 s. A total of 751 samples in two different sites in Germany could be collected in the year 2020.

### 2.2. Greenhouse Experiment

To increase the number of samples with a high range of infection, additional greenhouse (GH) beets were artificially inoculated. After 8 to 10 weeks of growth, each beet was inoculated with 0.25 g of infected, ground barley. The trial was harvested after 14 to 21 days post infection, depending on the infestation of the susceptible controls. The samples from the GH were rated on a single beet bases considering the percentage of infection. Each sample consisted of 10 infected single beets belonging to the same scoring class, which were, after the scoring, ground into a very fine pulp using a kitchen hand mixer. The resulting fine pulp was spread in 7-mm-thick plates and immediately measured by NIRS with the same spectrometer model and in the same way as described above for the harvest in the field. In total, 70 samples from the GH were added to the data set.

### 2.3. RRCR Disease Index

Based on the scoring obtained after assessing the disease, the rhizoctonia disease index (RDI) was then calculated as

$$\mathrm{RDI}=\frac{\sum \mathrm{disease}\;\mathrm{severity}\;\mathrm{class}\;\times \;\mathrm{nr}\;\mathrm{plants}\;\mathrm{within}\;\mathrm{that}\;\mathrm{class}}{\sum \mathrm{nr}\;\mathrm{plants}\;\mathrm{in}\;\mathrm{each}\;\mathrm{class}}$$

### 2.4. NIR Chemometrical Modelling

The disease stage of some rhizoctonia infected beets might be so advanced that harvest is not feasible anymore. Translated into sampling, this means that (i) samples in the high range of infection might be missing, and (ii) many more samples were scored in the low range of infection than in the higher range of infection, independently of how high the rhizoctonia infection was originally in the field. Point (i) led to the GH measurements described above. Point (ii) would have caused spectral redundancy in the lower infection scoring range. To overcome redundancy, a Gauss-Jordan algorithm was applied in order to select only informative spectra. From the 821 available samples, a resulting set of 370 spectra could be used for further chemometrical modelling. This set of informative spectra was split into a calibration and an independent validation set. To obtain a representative validation set, an algorithm was applied to the 370 samples set, selecting one in every five samples (74 samples in total). The calibration set consisted of the remaining 296 samples. Model development was based on Partial Least Squares Regression (PLSR), with full cross-validation as an internal validation method. The prediction accuracy of the models was tested with the above-described independent validation set and with an additional independent validation set comprising 94 samples from a field in Germany with natural Rhizoctonia infestation from harvest season 2021.

Several spectroscopic data pre-treatments were tested to optimize chemometrical modelling. Sequential combinations of first derivative, second derivative, normalization, standard normal variate, fixed first derivative, fixed second derivative, Savitzky–Golay and detrending were analysed. The parameters considered to select an optimal calibration model were a low Root Mean Square Error of Cross-Validation (RMSECV), low bias, low Standard Error of Prediction (SEP), low Root Mean Square Error of Prediction (RMSEP), high Ratio of Prediction to Deviation (RPD), and high Pearson correlation coefficient (R).

During the prediction model development, outliers were identified and removed by selecting samples with high residual values. Leverage outliers were calculated over all calibration set spectra using the *H* statistic according to the formula $E\left(H\right)=\frac{K+1}{n}$, where *E*(*H*) is the average value that indicates a measure of multidimensional distance of a spectrum to the regression line, *k* is the number of factors and *n* the number of spectra in the calibration set. Values higher than 3 were considered outliers. Spectrum reconstruction error outliers were obtained by recalculating the original spectrum from the selected factors. For that, the mean deviation of the reconstructed spectrum was calculated over all wavelengths and normalized to the mean deviation calculated over all calibration set spectra. Spectra with values higher than 5 were considered outliers. Outliers that are both leverage and spectral reconstruction were calculated through a student *t* test ($t_{i}=\frac{e}{SEE\;\sqrt{1-H}}$) and through a Cook’s statistic test ($D=\frac{t_{i}{}^{2}\cdot \;H}{\left(k+1\right)\cdot \;\left(1-H\right)}$), where *t_i_* corresponds to the residual error, *e* is the difference between the estimated value and the reference value, *SEE* is the standard error of estimation, *H* is the leverage statistic for this sample, *D* is the value indicating the influence of the spectrum in the regression model and *k* is the number of factors. Spectra with values higher than 3 were listed as outliers.

SensoLogic Calibration Workshop v.2.10 and SensoLogic Calibration Wizard v.3.0 software packages (SensoLogic GmbH, Norderstedt, Germany) were used for model optimization.

## 3. Results

**Figure 4:**
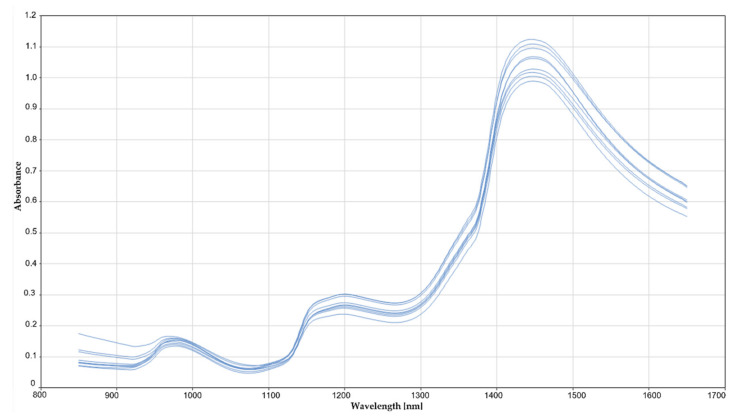
Example of eight NIR spectra from sugar beet pulp, corresponding to healthy and RRCR infected beets with different degrees of disease severity. The spectrometer was used in reflectance mode and data was transformed into absorbance.

**Figure 5:**
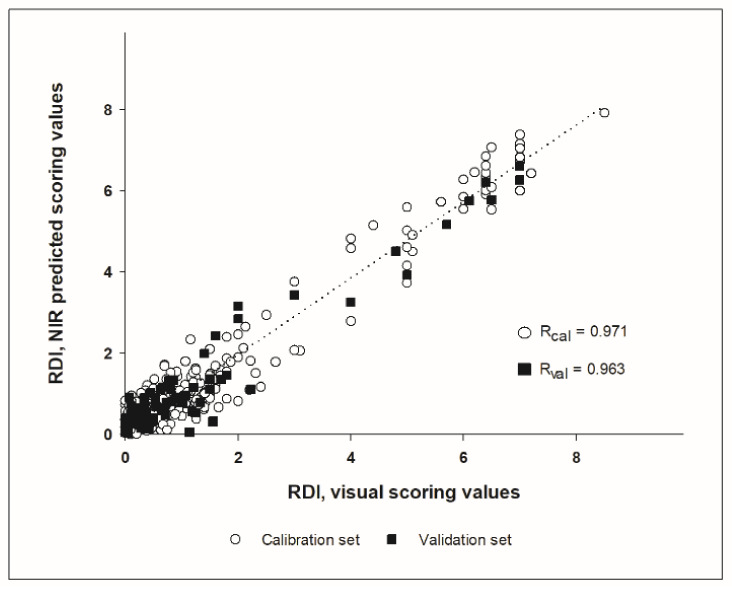
NIR calibration model for rhizoctonia detection in sugar beet beets using the rhizoctonia disease index (RDI). Calibration (*n* = 296) and validation samples (*n* = 74) are represented, as well as the Pearson correlation coefficients for both data sets (R_cal_ and R_val_).

**Figure 6:**
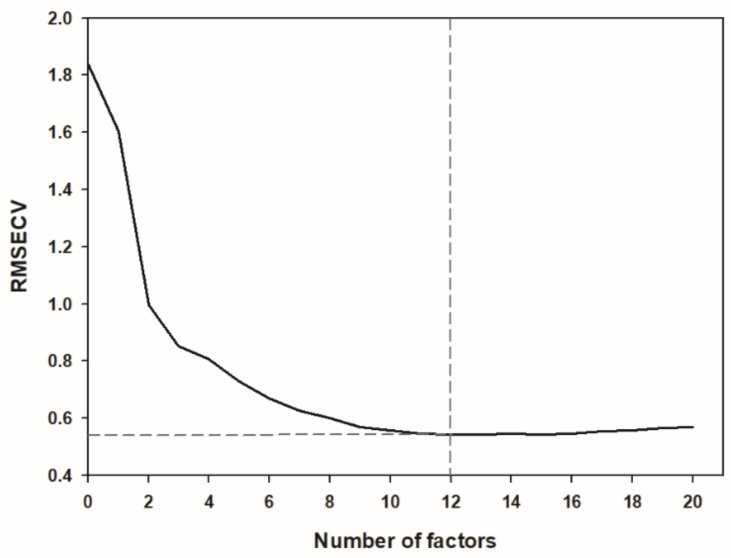
Number of factors and Root Mean Square Error of Cross Validation (RMSECV) obtained through the cross-validation process using a PLSR method. The number of factors considered optimum is indicated by the dashed lines.

The RDI scoring according to the formula described above ranged from 0 to 3.1 in field samples and from 0 to 8.5 in GH samples. A first visual screening of the spectra did not allow the detection of any differences between healthy or highly infected samples (Figure 4). The whole spectral range measured (850 to 1650 nm) could be used for calibration development, without the need to remove noisy bands at the spectral ends. The best result for RRCR quantification was obtained by applying a first derivative followed by a standard normal variate pre-treatment. Six samples (2% of the total calibration set) were considered leverage and spectral reconstruction outliers and were removed from the calibration set. The calibration model found optimal for RRCR quantification achieved a Pearson correlation coefficient of R = 0.972 (Figure 5) and a Ratio of Prediction to Deviation (RPD) of 4.131. The maximum number of factors calculated was 20, and the most advantageous number of factors for the optimized model was considered 12 (Figure 6). A decrease or an increase in the number of factors was worsening the prediction results.

**Figure 7:**
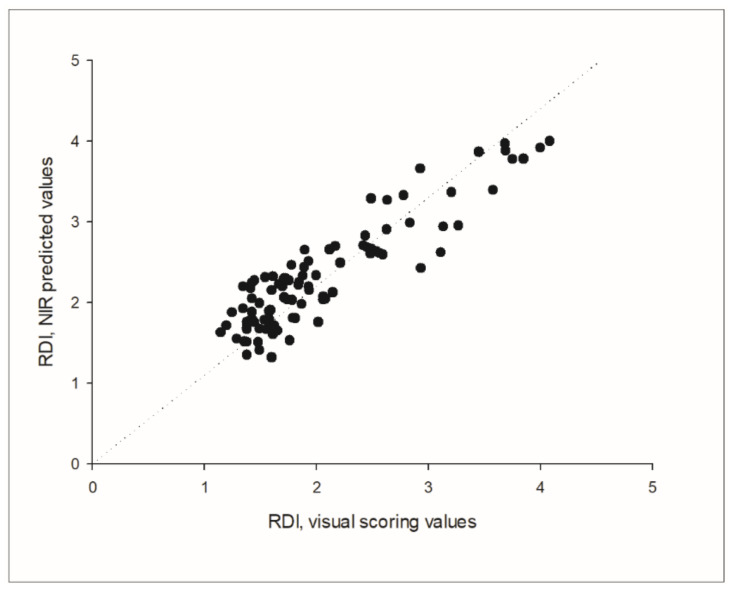
Prediction of an external independent data set obtained from a field with natural Rhizoctonia contamination from harvest season 2021 in Germany (*n* = 94; R = 0.901).

The prediction of the independent validation data set showed a good adjustment between the predicted NIR values and the visual scoring values, with a correlation coefficient of R = 0.963, which was found to be highly significant (*p* < 0.001), and a RPD of 3.677. The parameters standard error of prediction (SEP) and root mean square error of prediction (RMSEP) indicated a good predictive ability as well, since both SEP and RMSEP were equal up to the second decimal (0.497 and 0.494, respectively). Considering that the SEP squared is approximately equal to the RMSEP squared minus the bias squared, this was also reflected in the low bias of the prediction, that was −0.029 in absolute terms. The prediction of the external independent data set from harvest 2021 resulted in a correlation coefficient of R = 0.901, Bias = 0.283 and SEP = 0.308 (Figure 7).

## 4. Discussion

The results of this study show that the detection and quantification of RRCR directly in beets is feasible with NIR. To the best of our knowledge, this is the first study that uses NIR measurements directly on sugar beet root pulp to detect and quantify RRCR based on an incidence scoring in the field. Although the assessment of the disease severity using spectroscopy methods has been proved to be possible in sugar beet, most of these studies were either carried out on leaves or under controlled conditions [12,13,14,15]. As demonstrated by Scholten et al. [10], leaf and root symptoms often do not correlate, and the use of root symptoms proved to be more recommendable for disease assessment as they are more accurate. Moreover, despite the numerous advantages of GH testing, the necessity to test rhizoctonia directly in the field has been pointed out before [1,25]. Besides the fact that RCRR is affected by environmental conditions in a great measure [26], sugar beet genotypes react differently to different climate and soil conditions. Thus, the interaction genotype-environment can only be effectively evaluated in the field and not in a greenhouse.

The optimal calibration model was developed by using PLSR method as linear regression and for that, the number of factors used in the model is of crucial importance. The optimum number of factors (in this model considered 12) is usually the number where the Root Mean Square Error of Cross Validation (RMSECV) has its first minimum and is found through the cross-validation process, decomposing the spectral data matrix between the informative part of the spectral signature and the noise [27]. If the full set of factors is used (in our case 20), there is no clear distinction between the informative part of the spectral signature and the noise, thus generating an overfitted model that would include parts of the spectral noise of the calibration set spectra as prediction relevant variables. This would result in a poor performance in the validation set prediction despite of a low RMSECV. On the contrary, the use of too few factors would result in an underfitted model [28,29]. Thus, the number of factors here found optimal might be considered one of the indicators of the good fit of the prediction model.

Additionally, the coefficients of correlation in both calibration (0.972) and prediction (0.963) reflect the prediction accuracy, that is how close the prediction values are to the parameter values used as a reference. Normally, lab reference values are used for modelling, in this study a visual RRCR scoring was used instead, as this is the usual approach to score RRCR in the field nowadays. In this regard, it might be mentioned that a NIRS scoring could provide an objective advantage over potential biases of visual scorings.

Other parameters providing information about the prediction accuracy are the SEP, RMSEP and the bias. Ideally, predictions should be as accurate as possible, resulting in low RMSEP and SEP values [30]. The bias is a good indicator of similarity between validation samples and the calibration set [31], conveying a further measure of the goodness of the fit. In sum, the bias of −0.029 obtained in this application together with the SEP and RMSEP values indicate a good adjustment between the predicted NIRS values and the RDI visual scoring of the beets. Moreover, the high correlation observed between the NIR prediction and the RDI values from the external independent validation set argues against a possible overfitting of the model and corroborates the efficiency of this NIR application.

Another useful indicator in the development of calibration models is the RPD, which has been used for several years in NIR studies of agricultural products [32,33]. It enables the evaluation of the SEP in terms of the standard deviation of the reference data. The RPD values considered appropriate for each NIRS analysis may change depending on the nature of the material. Within agricultural applications, values higher than 3 may be difficult to obtain, and often models with RPD > 3.5 are considered very good [33,34]. Therefore, the RPD values of 4.131 in the calibration and 3.677 in the prediction found in this study corroborate the goodness of fit of the NIR predictions developed here.

It should be considered that any given calibration model might need to be regularly updated by adding further locations and harvesting years in order to maintain high robustness in the prediction of new samples. Hence, more data is still needed to assure a representative calibration model in the future. However, the results obtained here show the potential of NIR spectroscopy to detect RRCR directly in the beet pulp and can be interpreted as a promising starting point for extending the procedure to practical field applications. One important advantage is that disease quantification can thus be easily integrated and run in parallel with the harvest. Thereby, the complete harvested field can be scored, without any limiting sampling area. The NIR-based estimation of RRCR infection can thus be incorporated in the yield data analyses. RRCR estimation can be considered as a cofactor to enhance the precision and therefore, the heritability of the sugar yield per acreage. The more variation one can attribute to certain sources the less variation is in the error term and selection gain can be increased. The very easy handling and speed of the NIRS technique allows a fast objective scoring that can be automated, incorporated to a harvester machine and performed cost-effectively directly in the field without personnel capacity limits, human scoring bias, nor time restraints. Taken together, results suggest that this NIRS application might be a useful tool for breeding processes and selection of sugar beet resistant varieties.

## References

[1] Büttner G., Pfähler B., Märländer B. (2004). Greenhouse and field techniques for testing sugar beet for resistance to Rhizoctonia root and crown rot. Plant Breed..

[2] Liu Y., Qi A., Khan M.F.R. (2019). Age-dependent resistance to *Rhizoctonia solani* in sugar beet. Plant Dis..

[3] Märländer B., Hoffmann C., Koch H.-J., Ladewig E., Merkes R., Petersen J., Stockfisch N. (2003). Environmental situation and yield performance of the sugar beet crop in Germany: Heading for sustainable development. J. Agron. Crop Sci..

[4] Anees M., Edel-Hermann V., Steinberg C. (2010). Build up of patches caused by *Rhizoctonia solani*. Soil Biol. Biochem..

[5] Herr L.J. (1996). Sugar beet diseases incited by *Rhizoctonia* spp. *Rhizoctonia* Species: Taxonomy, Molecular Biology, Ecology, Pathology and Disease Control.

[6] Hillnhütter C., Albersmeier A., Berdugo C.A., Sikora R.A. (2011). Synergistic damage by interactions between *Ditylenchus dipsaci* and *Rhizoctonia solani* (AG 2-2IIIB) on sugar beet. J. Plant Dis. Prot..

[7] Windels C.E., Nabben D.J. (1989). Characterization and pathogenicity of anastomosis groups of *Rhizoctonia solani* isolated from *Beta vulgaris*. Phytopathology.

[8] Allen M.F., Boosalis M.G., Kerr E.D., Muldoon A.E., Larsen H.J. (1985). Population dynamics of sugar beets, *Rhizoctonia solani*, and *Laetisaria arvalis:* Responses of a host, plant pathogen, and hyperparasite to perturbation in the field. Appl. Environ. Microbiol..

[9] Buhre C., Kluth C., Bürcky K., Märländer B., Varrelmann M. (2009). Integrated control of root and crown rot in sugar beet: Combined effects of cultivar, Crop rotation, and soil tillage. Plant Dis..

[10] Scholten O.E., Panella L.W., De Bock T.S.M., Lange W. (2001). A greenhouse test for screening sugar beet (*Beta vulgaris*) for resistance to *Rhizoctonia solani*. Eur. J. Plant Pathol..

[11] Hillnhütter C., Mahlein A.K., Sikora R.A., Oerke E.C. (2011). Remote sensing to detect plant stress induced by *Heterodera schachtii* and *Rhizoctonia solani* in sugar beet fields. Field Crops Res..

[12] Hillnhütter C., Mahlein A.K., Sikora R.A., Oerke E.C. (2012). Use of imaging spectroscopy to discriminate symptoms caused by *Heterodera schachtii* and *Rhizoctonia solani* on sugar beet. Precis. Agric..

[13] Hillnhütter C., Sikora R.A., Oerke E.C. (2011). Influence of different levels of resistance or tolerance in sugar beet cultivars on complex interactions between *Heterodera schachtii* and *Rhizoctonia solani*. Nematology.

[14] Reynolds G.J., Windels C.E., MacRae I.V., Laguette S. (2012). Remote sensing for assessing *Rhizoctonia* crown and root rot severity in sugar beet. Plant Dis..

[15] Barreto A., Paulus S., Varrelmann M., Mahlein A.K. (2020). Hyperspectral imaging of symptoms induced by *Rhizoctonia solani* in sugar beet: Comparison of input data and different machine learning algorithms. J. Plant Dis. Prot..

[16] Strube GmbH & Co. (2016). Verbundvorhaben: Beschleunigte und Präzisere Züchtungsforschung Durch ein Nicht-Invasives Hochdurchsatz-Screeningsystem zur Frühzeitigen Erkennung von Pathogenbefall der Zuckerrübe—Teilvorhaben 1: Durchführung der Versuche, Biotests und Beprobung: Schlussbericht zum Vorhaben : Laufzeit: 01.09.2012 bis 31.08.2015.

[17] Al-Amery M., Geneve R.L., Sanches M.F., Armstrong P.R., Maghirang E.B., Lee C., Vieira R.D., Hildebrand D.F. (2018). Near-infrared spectroscopy used to predict soybean seed germination and vigour. Seed Sci. Res..

[18] Givens D.I., Deaville E.R. (1999). The current and future role of near infrared reflectance spectroscopy in animal nutrition: A review. Aust. J. Agric. Res..

[19] Pasquini C. (2018). Near infrared spectroscopy: A mature analytical technique with new perspectives—A review. Anal. Chim. Acta.

[20] Heppner S., Thielecke K., Buchholz K., Wullbrandt D. (2000). Potential applications of NIR spectrometry in the sugar industry. Zuckerindustrie.

[21] Huijbregts A.W.M., De Regt A.H., Gijssel P.D. (1996). Determination of some quality parameters in sugar beet by near infrared spectrometry (NIRS). Commun. Soil Sci. Plant Anal..

[22] Martínez-Arias R., Müller B.U., Schechert A. (2017). Near-Infrared Determination of Total Soluble Nitrogen and Betaine in Sugar Beet. Sugar Tech.

[23] Pan L., Lu R., Zhu Q., McGrath J.M., Tu K. (2015). Measurement of moisture, soluble solids, sucrose content and mechanical properties in sugar beet using portable visible and near-infrared spectroscopy. Postharvest Biol. Technol..

[24] Roggo Y., Duponchel L., Huvenne J.P. (2004). Quality Evaluation of Sugar Beet (*Beta vulgaris*) by Near-Infrared Spectroscopy. J. Agric. Food Chem..

[25] van Bruggen A.H.C., Grünwald N.J., Bolda M., Sneh B., Jabaji-Hare S., Neate S., Dijst G. (1996). Cultural methods and soil nutrient status in low and high input agricultural systems, as they affect *Rhizoctonia* species. Rhizoctonia Species: Taxonomy, Molecular Biology, Ecology, Pathology and Disease Control.

[26] Bolton M.D., Panella L., Campbell L., Khan M.F.R. (2010). Temperature, moisture, and fungicide effects in managing rhizoctonia root and crown rot of sugar beet. Ecol. Epidemiol..

[27] Xie L., Ye X., Liu D., Ying Y. (2009). Quantification of glucose, fructose and sucrose in bayberry juice by NIR and PLS. Food Chem..

[28] Gayo J., Hale S.A. (2007). Detection and quantification of species authenticity and adulteration in crabmeat using visible and near-infrared spectroscopy. J. Agric. Food Chem..

[29] Minaei S., Bagherpour H., Noghabi M.A., Khorasani Fardvani M.E., Forughimanesh F. (2016). A Comparative Study Concerning Linear and Nonlinear Models to Determine Sugar Content in Sugar Beet by Near Infrared Spectroscopy (NIR). J. Food Biosci. Technol..

[30] Downes G.M., Meder R., Hicks C., Ebdon N. (2009). Developing and evaluating a multisite and multispecies NIR calibration for the prediction of Kraft pulp yield in eucalypts. South. For..

[31] Igne B., Gibson L.R., Rippke G.R., Hurburgh C.R. (2007). Influence of yearly variability of agricultural products on calibration process: A triticale example. Cereal Chem..

[32] Bellon-Maurel V., Fernandez-Ahumada E., Palagos B., Roger J.M., McBratney A. (2010). Critical review of chemometric indicators commonly used for assessing the quality of the prediction of soil attributes by NIR spectroscopy. Trends Anal. Chem..

[33] Williams P. (2014). The RPD Statistic: A Tutorial Note. NIR News.

[34] Chang C.W., Laird D.A., Mausbach M.J., Hurburgh C.R. (2001). Near-Infrared reflectance spectroscopy-principal components regression analyses of soil properties. Soil Sci. Soc. Am. J..

